# Euglycemic Ketoacidosis and Lactic Acidosis Associated With Metformin Toxicity

**DOI:** 10.7759/cureus.60661

**Published:** 2024-05-20

**Authors:** Feras Al-Moussally, Jung-Jung Tien, Kanya Rajagopalan, Carlos Boterosuarez, Roger Crouse

**Affiliations:** 1 Department of Internal Medicine, University of Central Florida College of Medicine, Kissimmee, USA; 2 Department of Internal Medicine, University of Central Florida College of Medicine, Orlando, USA; 3 Department of Internal Medicine, University of Central Florida Hospital Corporation of America (HCA) Healthcare Graduate Medical Education (GME), Orlando, USA

**Keywords:** mellitus, metformin, euglycemia, hypoglycemia, diabetes type 2, diabetes

## Abstract

In patients with diabetes, diabetic ketoacidosis (DKA) is a well-documented potential complication, usually presenting with hyperglycemia, anion gap acidosis, and positive ketones. Metformin toxicity in the setting of acute renal failure is also a well-known cause of lactic acidosis. However, metformin-induced euglycemic ketoacidosis is less well-known or studied. We report a case of metformin toxicity in the setting of acute renal failure with both lactic acidosis and ketosis and an initial confounded clinical presentation of sulphonylurea-induced hypoglycemia. A high index of suspicion for metformin-associated lactic acidosis (MALA) and metformin-associated lactic acidosis with euglycemic ketoacidosis (MALKA) should be in place in patients who are taking metformin and presenting with acute renal failure and euglycemia.

## Introduction

As the first-line pharmacologic treatment, metformin is the most prescribed drug for type 2 diabetes. Metformin is a well-known cause of increased lactate levels. Metformin-associated lactic acidosis (MALA) has a mortality rate as high as 50% [1,2]. Metformin-associated lactic acidosis with euglycemic ketoacidosis (MALKA), on the other hand, is less reported in the literature and presents a more challenging diagnosis, necessitating a high index of suspicion for the primary team, especially in the presence of other pharmacological agents interfering with the presentation and laboratory results [3,4].

This case was previously presented as an abstract at the May 2022 American Association of Clinical Endocrinology meeting.

## Case presentation

We present a 67-year-old female patient with a past medical history of diabetes mellitus type 2, stage IV chronic kidney disease (CKD) at baseline with a GFR of 28, hypertension, atrial fibrillation on warfarin, and hypothyroidism who was brought to the emergency department by her daughter due to a report of falls, confusion, headaches, polyuria, as well as poor oral intake, nausea, and acute diarrhoea for approximately five days. Family members also noted a blood glucose reading of 50 mg/dL at home. The patient denied fever, chills, cough, vomiting, or rashes in the emergency department. Her home medications included metformin 500 mg twice daily, glimepiride 4 mg daily, glipizide 10 mg twice daily, warfarin 4 mg daily, levothyroxine 75 mcg daily, donepezil 10 mg daily, and quetiapine 60 mg daily. For many years, and one week prior to presentation, her primary care physician had started regular insulin 6 units with meals.

**Figure 1:**
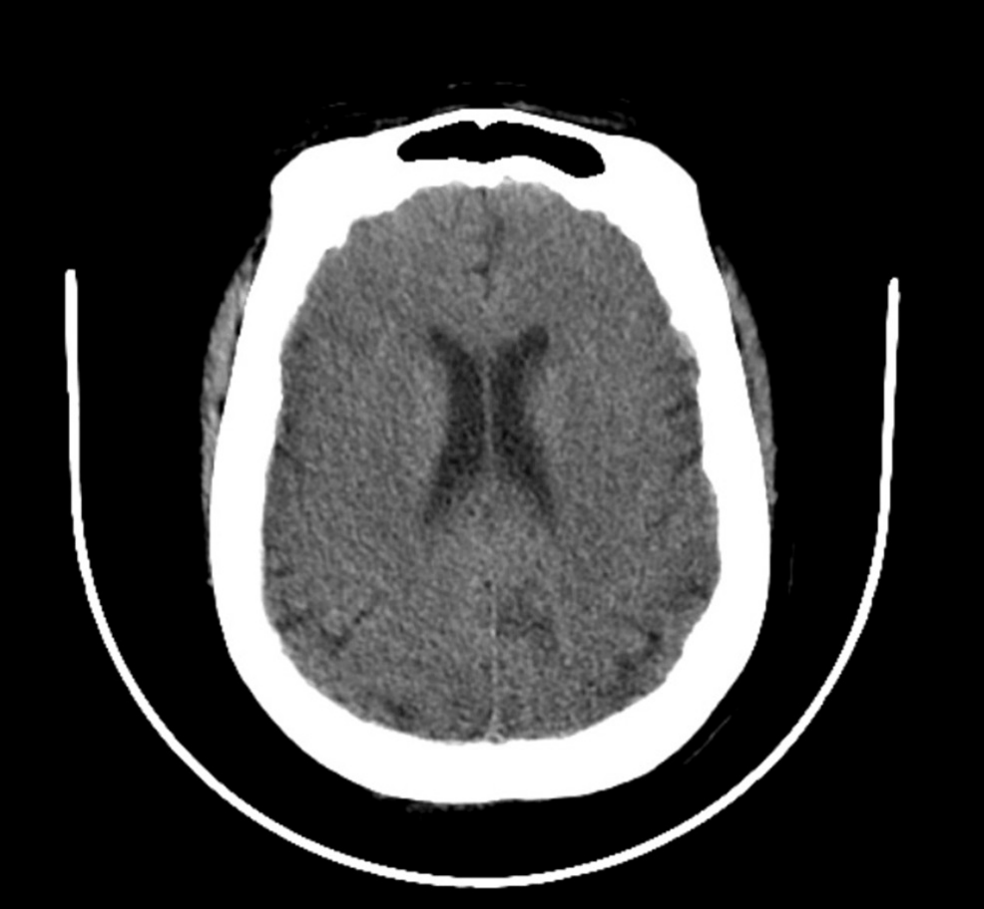
CT head

**Figure 2:**
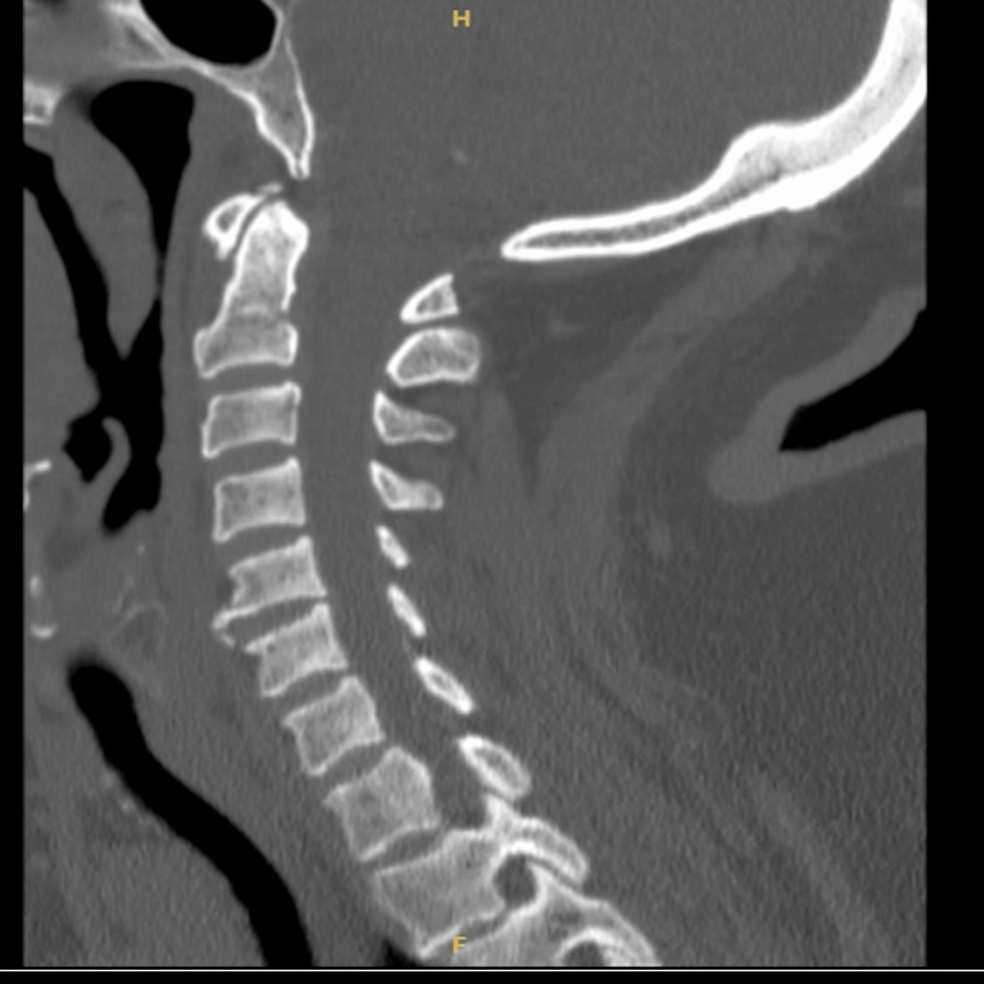
CT cervical spine

In the emergency department, she was found to have a temperature of 96.7° F, a heart rate of 75 beats per minute (bpm), a respiratory rate of 24 respirations per minute, and a blood pressure of 130/72 mmHg. She was initially alert, awake, confused, and disoriented, without any neurologic focalization, a normal sensory and motor exam, or a normal cardiovascular and respiratory examination. The initial workup showed a blood glucose of 53 mg/dL, and she was given 50 mL of 50% dextrose with subsequent hourly glucose values of 182, 198, and 185 mg/dL. Octreotide 50 mcg was subcutaneously initiated for suspected sulphonylurea-induced hypoglycemia as well as maintenance D5 + NS 125 mL/hr. A computed tomography (CT) of the head and cervical spine was performed and was largely unremarkable other than showing parietal scalp soft tissue swelling (Figures 1-2). The chest radiograph did not show any acute pathology. The electrocardiogram showed a sinus rhythm with left anterior fascicular block and signs of left ventricular hypertrophy with an R wave of 13mm in lead aVL, poor R wave progression with non-specific ST and T wave abnormalities in V4-V6, and QTC prolongation at 484. Initial labs were significant for bicarbonate of 11 mmol/L, creatinine of 9.43 mg/dL, blood urea nitrogen of 89 mg/dL, and glucose of 51 mg/dL (prior to D50) with a corrected anion gap of 27.3, a delta gap of 14, and a delta ratio of 1.1 suggestive of a pure high anion gap metabolic acidosis. INR was 1.3, and troponin was 0.03 ng/mL (reference range < 0.03 ng/mL). Nephrology was consulted for consideration of hemodialysis, given the severe acidosis.

During the process of admission, the patient unfortunately sustained a pulseless electrical activity (PEA) cardiac arrest in the emergency department, and return of spontaneous circulation (ROSC) was achieved in 13 minutes after five rounds of epinephrine and 300 mEQ of sodium bicarbonate. The suspected cause of the PEA was severe acidosis. The patient was intubated and started on mechanical ventilation with sedation. Arterial blood gas during the code showed pH 6.62, pCO_2_ 28 mmHg, pO_2_ 547 mmHg, HCO_3_ 2.9 mmol/L, and CO_2_ 11. CMP showed blood urea nitrogen 92 mg/dL, creatine 9.39 mg/dL, aspartate aminotransferase (AST) 287 units/L, alanine aminotransferase (ALT) 179 units/L, and calcium 9.9 mg/dL. Lactic acid was found to be 32 mmol/L (reference 0.4-2.0), and beta-hydroxybutyrate/acetoacetate was found to be 40.8 mg/dL (reference 0.2-2.8). She was promptly started on hemodialysis and the diabetic ketoacidosis (DKA) protocol with glucose and bicarbonate infusions. The high anion gap metabolic acidosis slowly improved with continuous veno-venous hemofiltration for 48 hours in order to treat lactic acidosis and metformin accumulation. CT abdomen with contrast ruled out mesenteric ischemic as a contributing cause of lactic acidosis. The patient had a hypoxic brain injury with the subsequent placement of a tracheostomy and a PEG tube seven days after intubation. A hospital sent out her blood level of metformin and subsequently proved her metformin toxicity level at 15 mcg/ml (normal therapeutic level: 0-2 mcg/ml).

## Discussion

In metformin-treated patients admitted for any emergency, there is a strong association with lactic acidosis when the metformin level is above 9.9 mg/L [5]. Renal replacement therapy (RRT) is the best modality to improve outcomes. In a population-based retrospective analysis of patients admitted with MALA undergoing RRT, the survival rate reached as high as 80% [6]. The etiology of metformin-induced lactic acidosis has been an area of interest for decades. Theories suggest that there are other confounding factors affecting and causing metformin-associated lactic acidosis, including, as in our case, circulatory collapse (type A lactic acidosis) and hypoxia [3,7]. The association of lactic acidosis with ketoacidosis in association with euglycemia is a much more formidable diagnosis, requiring a high level of suspicion on the clinician’s part. Metformin is not recognized as a cause for hypoglycemia, and when it occurs, it is usually in combination with other factors or drugs, such as sulfonylureas (e.g., glimepiride in our case) or the new class of SGLT2 inhibitors [8]. Metformin increases the transport of glucose into cells and decreases gluconeogenesis in the liver and kidneys by inhibiting the pyruvate dehydrogenase complex (PDC) through alteration of the redox potential and thus preventing the conversion of lactate and glycerol to glucose [9]. This creates a condition conducive to anaerobic metabolism despite adequate oxygen and leads to the increased metabolism of pyruvate into lactate. Further, renal impairment will result in a reduced clearance of both lactic acid and metformin, as well as creating another reason for acidosis, namely uremic acidosis. Thus, the increase in lactic acid can be viewed from both perspectives of lactate over-production (type A lactic acidosis) and decreased utilization of lactate by the liver (type B lactic acidosis). It is important to note that the pathophysiology of diabetic ketoacidosis is well known and is characterized by a relative or absolute lack of insulin and by an excess of counterregulatory hormones such as corticosteroids, glucagon, and catecholamines [10]. The counterregulatory hormones promote hyperglycemia by increasing gluconeogenesis, glycogenolysis, and decreased utilization of glucose. They also promote ketogenesis from free fatty acids through the processes of lipolysis in fat tissue and proteolysis of amino acids. In addition, in rats treated with metformin, it was shown that the treatment with metformin induced the production of ketoacidosis [6]. Glucose deficiency has a significant role in producing euglycemic diabetic ketoacidosis. The unabated excessive production of counterregulatory hormones will ensure the continued production of ketones, while the failure of gluconeogenesis can lead to a fall in blood glucose. We have already seen how metformin can inhibit gluconeogenesis even in the presence of counterregulatory hormones. Likewise, starvation and depletion of liver glycogen can result in lower-than-expected blood sugars. Increased glucagon also enhances lipid oxidation and the generation of acetyl-CoA and ketones when glucose is unavailable [11]. Metformin is generally not recommended in patients with a GFR<30 (CKD stages 4 and 5) [12]. But dose adjustments, specifically for our patient with CKD stage 4 at baseline, might be appropriate. According to Lalau et al., metformin 500 mg in the morning might be safe in CKD stage 4, with follow-up evaluation revealing appropriate stable metformin concentrations and the absence of hyperlactatemia (other than in patients who had a myocardial infarction) [13]. We believe that the best explanation is that our patient represents a case of lactic acidosis due to hypoxia, circulatory collapse with diabetic ketoacidosis due to extreme stress, and failure of gluconeogenesis due to metformin in the presence of acute renal failure.

## Conclusions

While this presentation is not common, we intended to make this case report to highlight pitfalls that could be avoided in patients presenting with hypoglycemia, an anion gap metabolic acidosis, while taking metformin. Pitfalls can include not taking into consideration renal function when adjusting the home diabetic regimen, failure to recognize ketoacidosis in the setting of euglycemia, and delays in the initiation of hemodialysis.
